# Mapping the space of protein binding sites with sequence-based protein language models

**DOI:** 10.1093/bioinformatics/btaf284

**Published:** 2025-06-27

**Authors:** Tuğçe Oruç, Maria Kadukova, Thomas G Davies, Marcel Verdonk, Carl Poelking

**Affiliations:** Computational Chemistry & Informatics, Astex Pharmaceuticals, Cambridge CB4 0QA, United Kingdom; Computational Chemistry & Informatics, Astex Pharmaceuticals, Cambridge CB4 0QA, United Kingdom; Computational Chemistry & Informatics, Astex Pharmaceuticals, Cambridge CB4 0QA, United Kingdom; Computational Chemistry & Informatics, Astex Pharmaceuticals, Cambridge CB4 0QA, United Kingdom; Computational Chemistry & Informatics, Astex Pharmaceuticals, Cambridge CB4 0QA, United Kingdom

## Abstract

**Motivation:**

Binding sites are the key interfaces that determine a protein’s biological activity, and therefore common targets for therapeutic intervention. Techniques that help us detect, compare, and contextualize binding sites are hence of immense interest to drug discovery.

**Results:**

Here, we present an approach that integrates protein language models with a 3D tessellation technique to derive rich and versatile representations of binding sites that combine functional, structural, and evolutionary information with unprecedented detail. We demonstrate that the associated similarity metrics induce meaningful pocket clusterings by balancing local structure against global sequence effects. The resulting embeddings are shown to simplify a variety of downstream tasks: they help organize the ‘pocketome’ in a way that efficiently contextualizes new binding sites, construct performant druggability models, and define challenging train-test splits for believable benchmarking of pocket-centric machine-learning models.

**Availability and implementation:**

A Python package that implements the EPoCS method is freely available at https://github.com/tugceoruc/epocs.

## 1 Introduction

Identifying protein-ligand binding sites and understanding their behaviour is crucial both for target discovery and structure-based molecular design (Anderson 2003, Ludlow *et al.* 2015). Common objectives are to detect cryptic subpockets (Meller *et al.* 2023), identify allosteric sites or protein–protein interfaces (Tubiana *et al.* 2022), assess druggability (Cheng *et al.* 2007), relate a site of interest to known pockets (Hedderich *et al.* 2022), transfer ligand information or hit matter across related sites (Eguida and Rognan 2020), understand selectivity and pharmacophoric constraints (Powers *et al.* 2023), or elucidate the effect of mutations (Wan *et al.* 2021). In practice, many of these analyses rely on techniques that efficiently search large databases such as the PDB for information on binding sites using appropriate filtering and ranking heuristics—i.e. *similarity metrics* (Ehrt *et al.* 2018, Eguida and Rognan 2022).

Many techniques that quantify pocket–pocket similarity have been proposed (Shulman-Peleg *et al.* 2005, Kuhn *et al.* 2007, Schalon *et al.* 2008, Yeturu and Chandra 2008, Konc and Janezic 2010, Wood *et al.* 2012, Desaphy *et al.* 2013, Gao and Skolnick 2013, Chartier and Najmanovich 2015, Simonovsky and Meyers 2020, Pallante *et al.* 2024), with significant differences in scope, focus, and speed. Typical is the use of both structural and physicochemical properties to describe residues, interactions and/or the pocket surface or shape. Some techniques are ligand-specific (i.e. they use a known ligand to bias the results; Wood *et al.* 2012, Desaphy *et al.* 2013, Chartier and Najmanovich 2015), others ligand-agnostic (Shulman-Peleg *et al.* 2005, Schalon *et al.* 2008, Yeturu and Chandra 2008, Konc and Janezic 2010, Gao and Skolnick 2013, Simonovsky and Meyers 2020). Faster approaches typically sacrifice sensitivity and accuracy for the ability to run queries interactively on the fly.

Despite this apparent abundance of approaches, we argue that more work on the topic is needed, and motivate this with the following three disparate observations:

- Transferable metrics that strike the ‘right’ balance between structural, physicochemical, sequence, and functional components remain a challenge.
- Protein-language models produce expressive vector embeddings that capture functional, evolutionary, and structural relationships simultaneously and with low computational cost (Bepler and Berger 2021, Ofer *et al.* 2021).
- Pocket-centric machine-learning models (e.g. for binding affinity or druggability prediction) are still often benchmarked with inappropriately debiased train-test splits that can produce unrealistic performance estimates (Buttenschoen *et al.* 2024).

The last point highlights that expressive similarity metrics that bridge multiple scales of information have applications beyond traditional target discovery and structure-based design. It is well established that debiased train-test splits are a prerequisite for believable benchmarks that enable fair comparisons while providing accurate estimates of a model’s prospective performance. Still, issues such as memorization and poor transferability of deep-learning architectures remain persistent, as highlighted by the recent PoseBusters study (Buttenschoen *et al.* 2024). And yet, despite this and other important efforts to establish best practices for validating deep-learning models, the proposed validation sets still often reduce to simple chronological splits that are prone to data leakage and use sequence identity cutoffs as a stand-in for more sophisticated 3D structural debiasing.

In this work, we combine 3D structural processing with protein-language models to construct ESM-driven Pocket Cross-Similarity (EPoCS)—a transferable, robust, general-purpose similarity metric for protein binding sites. We show that EPoCS successfully captures information at multiple scales, from function, to sequence, to local structure, while enabling visual queries and on-the-fly context-matching across the pocketome (Kufareva *et al.* 2012). By combining EPoCS with a hierarchical clustering approach, we debias train-test splits for improved benchmarking of machine-learning models with tunable sequences of progressively harder splits. We illustrate the approach for the prediction of protein druggability, highlighting how splits based on conventional homology cutoffs result in accidental data leakage, leading to less challenging validation sets than can be achieved with EPoCS.

The paper is organized as follows: Section 2 describes the formalism that integrates protein-language models with 3D tessellation techniques. Here, we also study the relationship and correlation with existing similarity metrics. Section 3 focuses on the visualization and key characteristics of EPoCS in organizing the pocketome and modelling local and global pocket-pocket cross-similarity. Section 4 describes our case study of applying EPoCS-derived train-test splits to druggability modelling.

## 2 Pocket cross-similarity

This section describes how we combine protein-language models, specifically, ESM-2 (Lin *et al.* 2023), with a 3D tessellation approach to construct a multiscale metric for pocket cross-similarity. We refer to this metric as EPoCS.

Protein language models (PLMs) have made dramatic progress over recent years, fuelled by the development of transformer-based architectures such as BERT (Devlin *et al.* 2019). They are typically trained using unsupervised or self-supervised protocols that task the model with reconstructing individual masked amino acids given an input sequence. PLMs have been applied successfully to a variety of problems, including the prediction of protein structures (Lin *et al.* 2023), mutation effects (Cheng *et al.* 2023), antibody design (Hie *et al.* 2024), and binding-site detection (Carbery *et al.* 2024). The fact that PLMs are so versatile points to how information-rich their learnt embeddings are, and how their implicit biochemical, functional, evolutionary, and chemogenomic information content is an emergent property of the pretext task that the models are subjected to during training.

Just how well these models integrate with structural applications is best demonstrated by ESMFold (Lin *et al.* 2023), a protein structure prediction model that substitutes AlphaFold2’s EvoFormer with an embedding layer derived from an ESM language model. However, even on its own, attention patterns of the ESM model have been shown to correlate well with real-space contacts of amino acids in the folded structure. This is an encouraging observation that justifies why ESM embeddings should also be a reasonable foundation for binding-site representations, where simultaneous descriptions of functional, structural, and evolutionary factors should prove beneficial.

**Figure 1.**
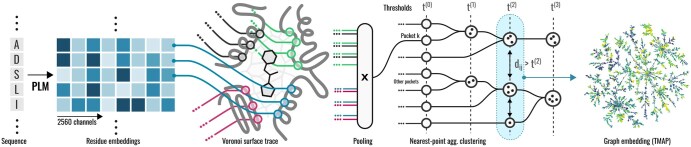
EPoCS approach for binding site representation and clustering. We use ESM-2 to generate embedding vectors for all residues of an input sequence. These embeddings are mapped onto the 3D structure of a protein–ligand complex. Radially truncated tessellation around a probe ligand identifies pocket interface residues of interest. Pooling of the embedding vectors over the interface results in the EPoCS embedding vector. The EPoCS metric is based on pairwise Euclidean distances between embedding vectors. Clusterings are obtained using an agglomerative strategy with strictly enforced minimum separations between clusters. We use tmap for visualization of the metric space.

Figure 1 summarizes the EPoCS approach that derives such representations from PLM embeddings. Given an input protein sequence, we use the 3B-parameter version of ESM-2 to generate embeddings for every residue in the sequence. These embeddings are then mapped onto an experimental 3D complex of the protein with a reference ligand. A radially truncated Voronoi tessellation (Barber *et al.* 1996, Olechnovič and Venclovas 2017) around the ligand atoms relative to backbone and side-chain atoms of the protein is used to identify the surface residues associated with the pocket volume. For nonliganded sites, a *pseudo*-ligand grown artificially into the pocket volume can serve as a substitute, even though this is not explored further in this study. The tessellation approach ensures that only surface-exposed residues that can form ligand interactions are included in the representations, thus reducing noise in the embeddings introduced by buried amino acids that carry little additional information content.

Mean pooling of the embedding vectors over the surface residues then gives rise to the EPoCS embeddings vector. The Euclidean distance between embedding vectors is used to measure the distance between binding sites and thus serves as input for further processing, e.g. for hierarchical clustering and dimensionality reduction (see Section 3). Note that using the 15B-parameter ESM-2 version produces quantitatively similar embeddings with Pearson and rank correlation coefficients for site-site distances of 0.98 and 0.96, respectively—see Supplementary Fig. S4. Given this similar behaviour, we opted for the 3B-parameter model due to its smaller computational footprint. Please also refer to the Supplementary Data for further details on processing of the embeddings, the tessellation approach, clustering, and 2D graph projection.

**Figure 2.**
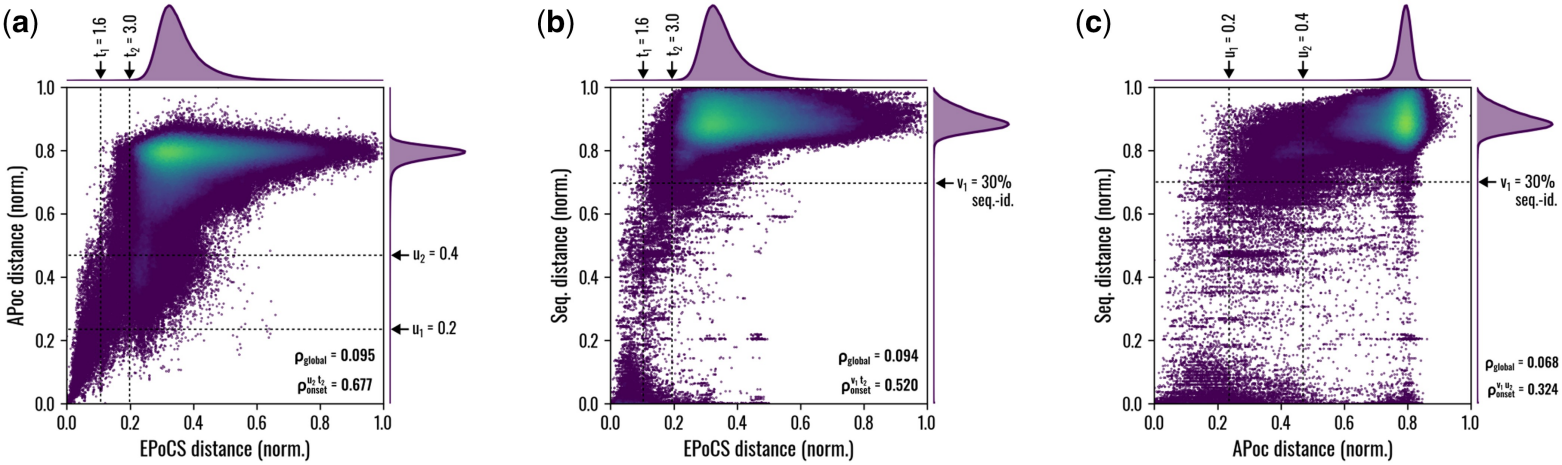
Correlation between EPoCS versus local (APoc) and global (sequence distance) baselines. Pairwise, normalized pocket–pocket distance correlations observed for (a) EPoCS versus APoc, (b) EPoCS versus sequence, (c) APoc versus sequence for a random set of 3500 binding sites from the PDB. EPoCS correlates well with both baseline metrics for pairs that the other measures deem similar, as quantified by an onset Spearman’s rank correlation coefficient $\rho_{\text{onset}}$ of 0.677 against APoc and of 0.520 against sequence distance. Here, the onset region is defined to contain all data points that fall below *either* of the maximum thresholds ($t_{2}$, $u_{2}$, $v_{1}$) indicated in each graph. The global correlation $\rho_{\text{global}}$, on the other hand, is very weak, not exceeding 0.1 for any of the three cases. For the baselines (c), the onset correlation of 0.324 falls significantly below the observations for EPoCS in panels (a) and (b). This points to the multiscale character of EPoCS that helps strike a balance between local structural, functional, and global evolutionary factors. Note that the thresholds $t_{1,2}$, $u_{1,2}$, $v_{1}$ referred to during the analysis (see main text) are labelled with their native (as opposed to min–max normalized) values.

We argue that EPoCS is an intrinsically multiscale representation of a binding site due to its direct link to ESM embeddings via interface-based tessellation. To demonstrate this, we compare the metric space induced by EPoCS to two limiting cases: First, Alignment of Pockets (APoc)—a computationally demanding metric based on local residue features and local pocket alignments (Gao and Skolnick 2013); second, a simple sequence distance measure. We compile a random set of ∼3500 pockets from the PDB (here referred to as PDB-3.5k) and measure all pairwise distances between the pockets using these three different techniques. The resulting correlation plots are shown in Fig. 2a–c. Interestingly, EPoCS produces a reasonable rank correlation $\rho_{\text{onset}}$ with both APoc (panel a, $\rho_{\text{onset}}=0.677$) and sequence distance (panel b, $\rho_{\text{onset}}=0.520$). Here, the onset region is defined to contain all data points that fall below *either* of the maximum clustering thresholds $t_{2},u_{2},v_{1}$ indicated in the graphs. The global correlation is weak, not exceeding 0.1 for any of the metric pairs. For the baselines, already the onset correlation shows pronounced scatter at $\rho_{\text{onset}}=0.324$ (c). Still, even a rank correlation of 0.677 as measured for EPoCS versus APoc suggests a sizable systematic differences between the metrics. For example, there is a small fraction of pairs for which APoc predicts very high similarity (distance <0.1) but that have an EPoCS distance above $t_{2}=3.0$. For the PDB-3.5k set, however, the fraction of these violations relative to all pairs with APoc distance <0.1 is just 0.14% and therefore of little concern. Comparing the marginal distributions generated by the individual metrics, EPoCS produces a better contrast across its distance domain with a heavier tail in the distance distribution, as opposed to the sharply peaked distributions of the baselines. This is further indication that EPoCS captures both the local, regional, and global similarity structure across the set, as will become even clearer when we visualize the metric space using projection techniques (Section 3).

As an advantage over other structure-based techniques such as APoc, EPoCS is alignment-free and thus computationally cheap. Applying APoc brute-force to a dataset such as the PDB would be unappealing due to the significant computational costs involved, as well as the fact that a large number of distant pocket–pocket pairs fall into a regime where APoc no longer produces any meaningful contrast. We can, however, compare the clustering outcome on this smaller subset of 3500 structures using concepts from optimal transport (OT) (Flamary *et al.* 2021): i.e. we establish a best-match assignment between the clusters induced by EPoCS to those induced by APoc for clustering thresholds *t* where the total number of clusters is comparable. The OT similarity between the clusterings is then defined as the average of the Jaccard index over the best-matching pairs of clusters. The results illustrate (Table 1) that EPoCS and APoc have on average a high agreement of 74%–86% between the best-matching cluster pairs, depending on the choice of threshold and exclusion of singleton clusters. This level of agreement indicates that EPoCS locally reproduces alignment-based techniques at a drastically reduced computational cost, while maintaining regional relationships across increasingly structurally decorrelated clusters.

**Table 1.** Cluster structure comparison between EPoCS and APoc.

Subset	EPoCS threshold	APoc threshold	OT cluster similarity	OT cluster similarity
	*t* / n_clusters	*u* / n_clusters	Including singletons	Excluding singletons
PDB-3.5k	1.6 / 1917	0.2 / 1938	0.86 (0.48)	0.74 (0.29)
PDB-3.5k	2.6 / 1407	0.3 / 1435	0.80 (0.35)	0.74 (0.25)
PDB-3.5k	3.0 / 1180	0.4 / 1119	0.75 (0.27)	0.75 (0.21)
Binding MOAD	1.6 / 8932	n/a / 7688	0.70 (0.23)	0.72 (0.18)

Clustering outcomes are compared for two different datasets: (i) a random subset of 3500 pockets from the PDB (PDB-3.5k) and (ii) the intersection of PDB-3.5k with Binding MOAD. Clustering thresholds *t* for EPoCS and APoc are adjusted so as to approximately match the number of induced clusters. The clustering similarity is evaluated using OT, and defined as the average of the Jaccard index of the best-matching pairs of clusters. The values in brackets are the null similarity index calculated for scrambled clusterings, where cluster sizes were retained, but the pocket labels randomly reshuffled across all clusters. The high agreement between 74% and 86% indicates that the EPoCS metric is locally consistent with alignment-based techniques.

## 3 Pocket Atlas

With EPoCS as similarity kernel, we can use well-established clustering and dimensionality reduction techniques to visualize the metric space and study its behaviour in more detail. The resulting clusterings and visualizations are also of practical interest, for example as a basis for pocket-focused search engines, ligand transfer, selectivity analyses, or (as discussed in Section 4) debiasing of train-test splits for validation and benchmarking of ML models.

Here, we use distance-based hierarchical clustering (Fig. 1) to group pockets into similarity clusters (Nielsen 2016). Hierarchical clustering is attractive as the agglomeration function allows us to enforce a strict minimum separation between clusters at varying thresholds *t* of the distance measure: i.e. given two distinct clusters *A* and *B*, with pockets a∈A and b∈B, the distance $d_{ab}$ is at least *t*. Even though the clustering is thus performed on a principled basis, we should keep in mind that the EPoCS metric itself remains a heuristic, and that *t* therefore needs to be selected empirically, with larger values of *t* resulting in fewer but more diverse clusters.

**Figure 3.**
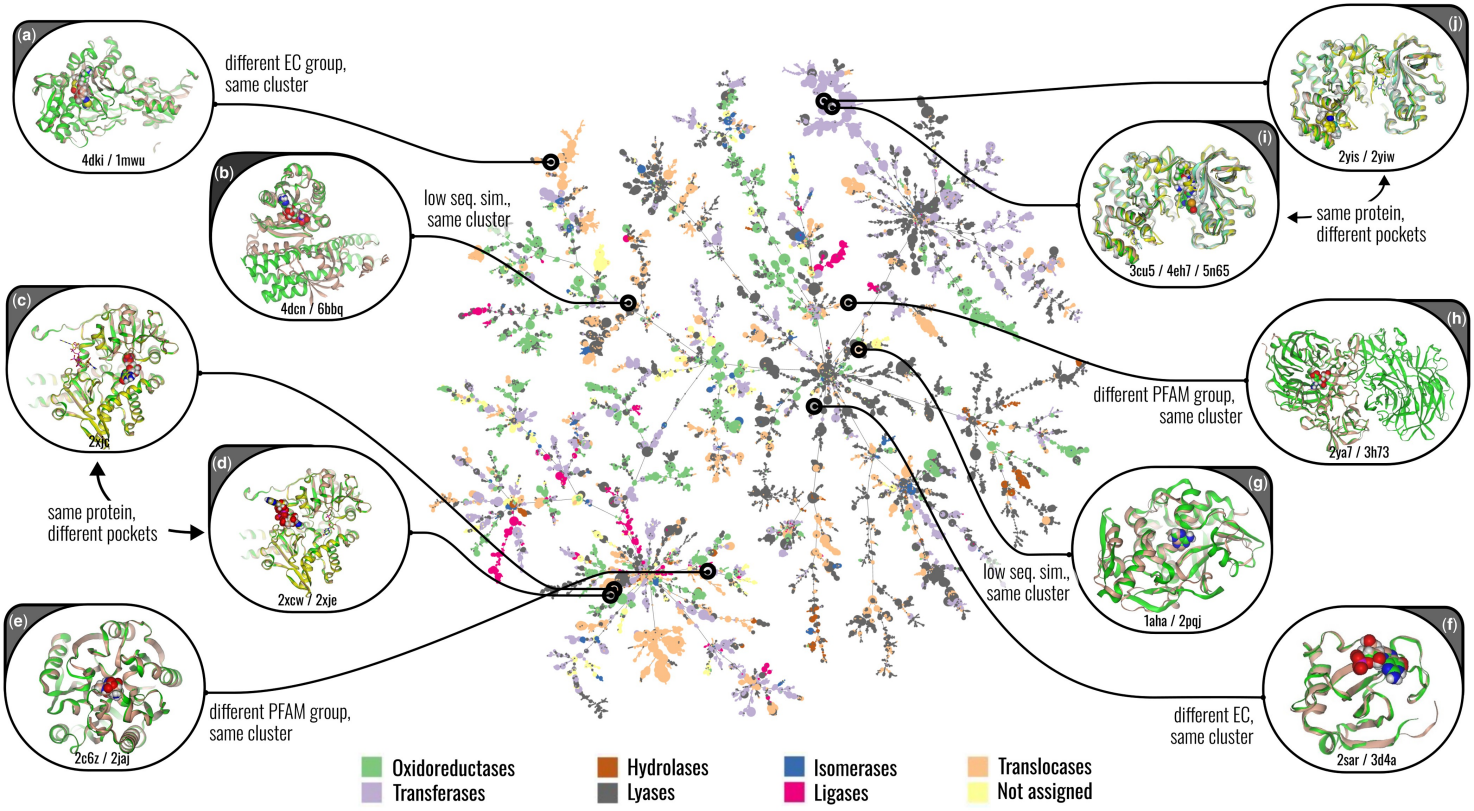
Pocket Atlas. Cluster projection produced by EPoCS for a set of 96k binding sites from the PDB. Each point corresponds to a cluster of pockets, scaled by cluster size. Clusters are coloured according to EC class if all members conform to the same functional label. Detailed inspection reveals that EPoCS reproduces meaningful structural and functional relationships both within and between clusters. Several examples are highlighted to illustrate that: (a, e, f, h) pockets from proteins with different EC or PFAM labels can co-cluster if they are structurally alignable; (c, d, i, j) spatially separated binding sites on the same protein fall into different but nearby clusters; (b, g) sites on proteins with low sequence similarity (<20%) can cluster together if they are structurally similar. See Supplementary Table S1 for details on the highlighted complexes. See the EPoCS GitHub page for a link to an interactive version of this map.

Figure 3 shows the cluster map for a curated set of 96k pockets extracted from 68k PDB structures at an EPoCS threshold of t=1.6 (see the Supplementary Data for details on the dataset curation). The 2D projection was generated using the Mapper algorithm, a technique from topological data analysis (see https://github.com/reymond-group/tmap). Each point on the map corresponds to a cluster of pockets, is scaled according to cluster size, and coloured by functional type (EC class). The links between clusters indicate the minimum spanning tree as weighted by the minimum distance of approach between the clusters. Statistics on the cluster-size distribution and EC-class fragmentation are shown in Supplementary Fig. S1.

In the following we provide evidence that this Pocket Atlas organizes the pocketome in useful ways: locally, by enforcing structural uniformity within each cluster; regionally, by linking clusters that are biologically or functionally related without an immediate structural homology.

**Figure 4.**
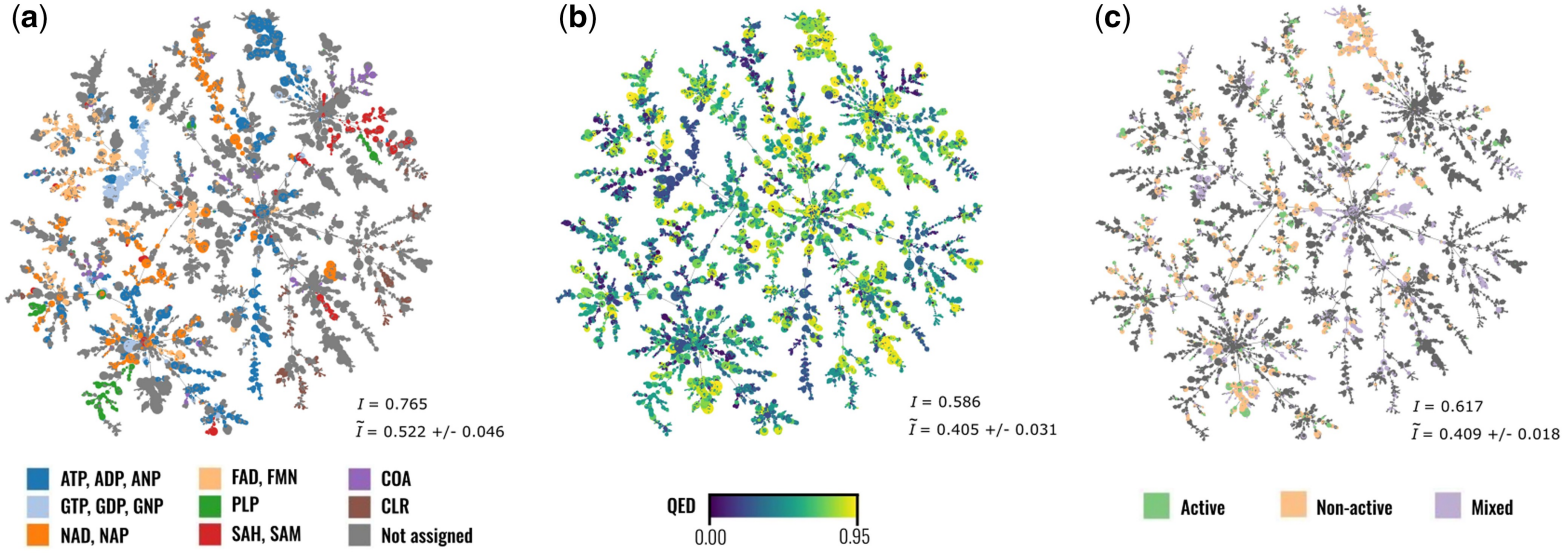
Chemical and functional coherence across EPoCS clusters. EPoCS maintains biochemical relationships locally and regionally. The colour maps indicate: (a) common ligands and cofactors, (b) drug-likeness (QED) scores, (c) active/catalytic site annotations. Spatial correlation coefficients (Moran’s *I*) evaluated using inter-cluster distances are stated in the bottom-right corner of each plot. $\tilde{I}$ is the null correlation estimated from random permutations of the property labels. See the Supplementary Data for details on processing of the labels and the definition of Moran’s *I*.

We begin by mapping relevant chemical and biological information onto the projection. Figure 4 shows colour maps for: (i) common ligands, (ii) drug-likeness (QED) scores, and (iii) UniProtKB active-site annotations. All three maps display significant local and global structuring, highlighting that EPoCS: (i) aligns well regionally with functional relationships by grouping catalytic sites in consistent ways; (ii) reproduces the well-known fact that similar environments bind similar ligands, but also that some ligands or cofactors (such as ATP) bind to diverse pocket environments—resulting in delocalized hotspots on the map; (iii) captures drug-likeness trends globally, as a likely consequence not just of the different levels of the ‘true’ druggability of the binding-site clusters, but also the intrinsic biases built into the PDB—with some targets and target classes receiving significantly more attention from the drug-discovery community than others.

Regarding the local cluster structure, one point of concern was that EPoCS could overemphasize the total protein sequence in the local site embeddings, thus potentially clustering together spatially separated sites on the same protein when they are structurally and functionally unrelated. This does not seem to be the case, however, with EPoCS instead allocating these sites to clusters that reside in the same region of the map, but are nevertheless kept well separated by the metric (see examples c, d, i, and j in Fig. 3). There are other examples that illustrate that EPoCS seems to conserve many local and regional relationships that are desirable to find in a Pocket Atlas. For example, different EC and PFAM groups can reside in the same cluster if their local structures align well. Even pairs of pockets from proteins with very low sequence identity (<20%) can fall into the same cluster if they are structurally related. This again highlights that EPoCS maintains high structural sensitivity despite its origin in sequence-based language models.

## 4 Pocket debiasing

Estimating the prospective performance of an ML model is hard, and it is not uncommon that models fail dramatically in a real-world setting despite performing well in benchmarks. This disconnect between benchmarking and prospective performance can result not only in a poor model being deployed, but also, potentially, the wrong model: specifically, one that beats the competition on a benchmark and produces good metrics by exploiting accidental bias in the data.

This problem is also pervasive in the development of pocket-centric ML models for protein-ligand docking, affinity, or druggability prediction (Durairaj *et al.* 2024). Here, the difficulty in evaluating and comparing diverse methods is partially due to the fact that the PDB (as the data source on which these models are directly or indirectly trained) is a heterogeneous database that was never designed with the training of ML techniques in mind: Its composition is heavily skewed towards certain targets and target classes while containing many ligands/cofactors and/or proteins that are biologically but not therapeutically interesting. Both these biases necessarily persist for random splits of PDB-derived data, as well as the chronological splits that seem to have become an accepted standard.

**Figure 5.**
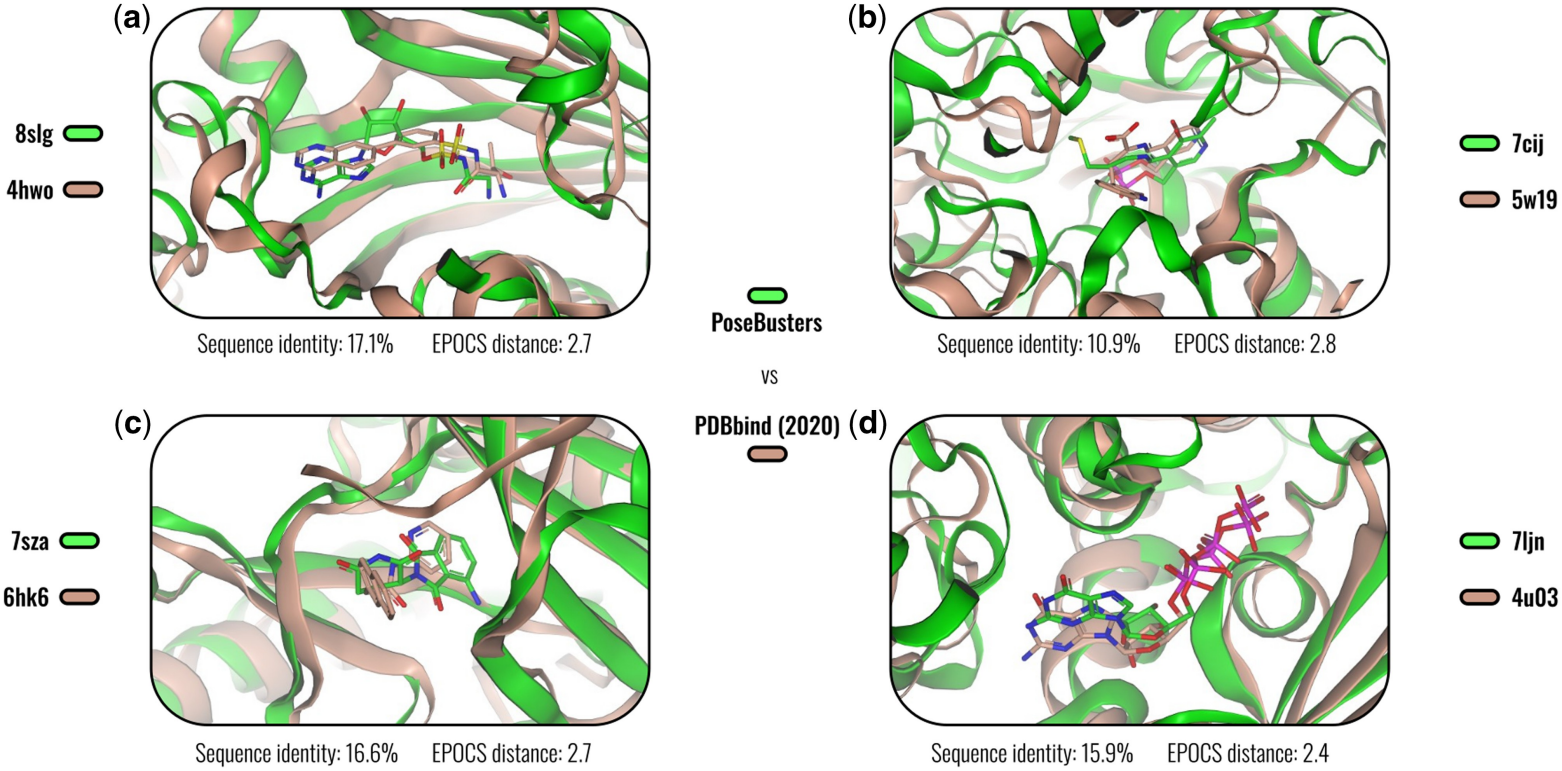
Low-sequence identity, high binding-site similarity. Examples that illustrate accidental overlap between the PoseBusters set and PDBbind 2020 for protein–ligand complex pairs with low sequence (<20%) but high binding-site similarity. Examples like these can potentially erode benchmarks by rewarding memorization on seemingly debiased validation sets. We used the EPoCS Pocket Atlas to co-project the low-homology PoseBusters and PDBbind set and thus identify representative examples as shown here. Sequence identities were computed with EMBOSS Needle. See Supplementary Table S1 for details on the complexes. Note that the complex pairs have an EPoCS distance below t=3.0, which is the largest clustering threshold considered in this work. Appropriate choices for the clustering threshold generally depend on the application, with train-test splits ideally based on larger, and visualizations such as those in Figs 3 and 4 on smaller thresholds.

Sequence-based splits try to improve on this practice, but still fail to prevent accidental data leakage between test and training sets. This is highlighted in Fig. 5, where we used the Pocket Atlas from Section 3 to identify examples in the PoseBusters set (a subset of PDBbind) where chronological and sequence-based splits still resulted in accidental overlap between the PDBbind training and PoseBusters validation set. Such overlap needs to be taken seriously given that ML models are second to none in memorization, and if there is a simple way to mitigate the issue, then we should make use of it.

Here, we show that EPoCS can provide us with one such simple strategy that efficiently decouples train-test splits for pocket-centric ML models. Moreover, we can achieve fine-grained control over the level of debiasing so as to create a sequence of progressively harder train-test splits. Conceptually, there are two different types of splits that can be generated with EPoCS: cluster-based splits, where we adjust the distance threshold *t* and then randomly allocate the resulting clusters to either the train or the test set; and tree-based splits, where we remove entire branches of the minimum spanning tree from Fig. 3 to create a challenging test of a model’s ability to generalize to entirely new pocket and protein contexts. We note that PLINDER (Durairaj *et al.* 2024) also uses graph-based approaches to define its debiased train-test split. The underlying similarity metric, however, relies on pairwise alignments and multiple independent measures of similarity capturing protein, pockets, and interactions, whereas EPoCS treats these summarily within a unified framework and in an alignment-free fashion. A comparison between PLINDER and EPoCS splits reveals that the induced train-test separation is qualitatively and quantitatively similar, see Supplementary Fig. S5.

We illustrate the two EPoCS debiasing strategies (cluster- versus tree-based) for the training and evaluation of a simple druggability model (referred to as ESM-MLP) that uses ESM-2 embeddings to predict druggability labels on a per-residue basis. Similar to the IF-SitePred model developed by Carbery *et al.* (2024), this model is trained on protein–ligand complexes with binary labels y∈{0,1} assigned to every residue of every complex depending on whether the residue is contacted by a ligand atom (y=1) or not (y=0). The cluster- and tree-based splits are constructed such that the training and test sets remain approximately constant in size as the threshold parameter *t* and test branches are varied.

**Figure 6.**
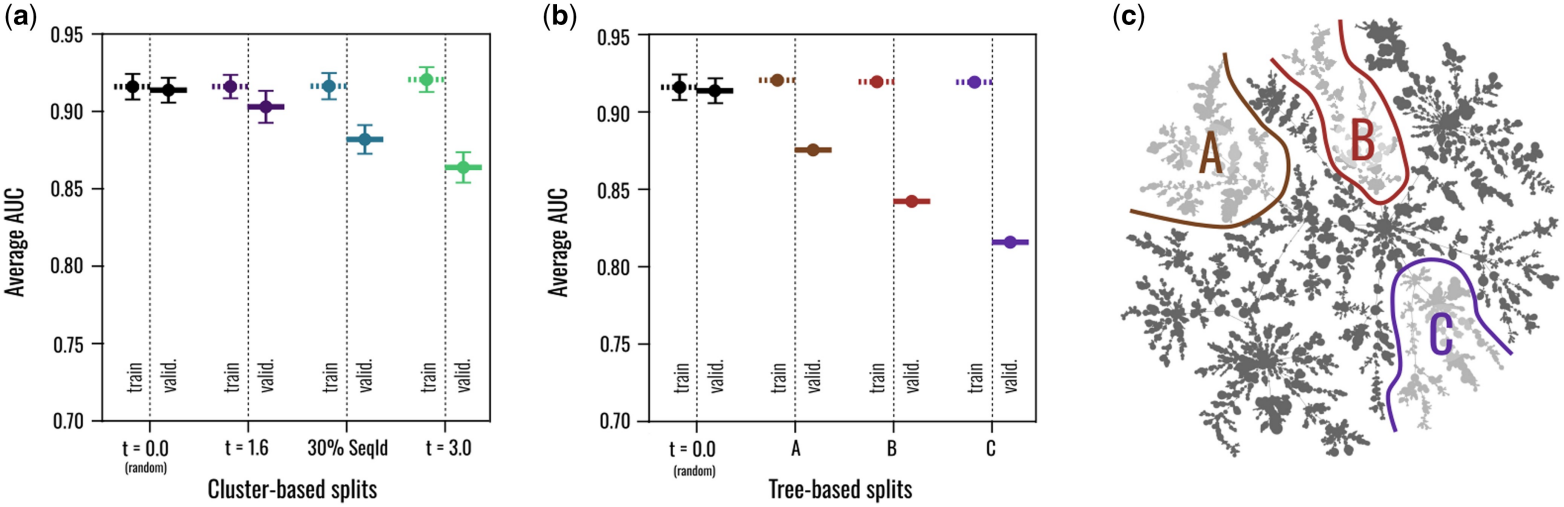
Progression of debiased train-test splits generated using EPoCS. Performance metrics of a pocket-centric ML model (here: for druggability prediction) obtained for different cluster- and tree-based splits generated using the EPoCS metric. Dashed and solid bars indicate training and test set performance, respectively. By varying (a) the threshold parameter *t* for cluster-based splits and (b and c) the test branch for tree-based splits, we obtain a sequence of progressively harder test sets designed to challenge the model, with the classification AUCs for the per-residue druggability scores decaying from >0.92 for a random split (t=0) to 0.81 for the hardest tree-based split (*C*). A homology-based split with a sequence-identity cutoff of 30% falls in between the cluster-based splits at t=1.6 and 3.0. The error bars indicate the standard error in cases where multiple instantiations of the same split protocol were tested. Note that the absolute sizes of the different train and test sets were kept approximately constant across all splits.

We note that ESM-MLP is performant compared to established druggability baselines including fpocket and PointSite (see the Supplementary Data for details). Here, however, we focus on the properties and behaviour of the splits and split progressions. The areas-under-the-curve (AUCs) for the different training and test sets are summarized in Fig. 6a–c. Note that here we have also added a homology-based split with a 30% sequence identity cutoff for comparison. For the cluster-based splits, we explore three different thresholds t=0,1.6,3.0, where 0 corresponds to a fully random split based on singleton clusters. We report test-train averages over three independent instantiations of those splits. For the tree-based splits, we consider three sections *A*, *B*, *C* of the pocket tree as test sets, as highlighted in Fig. 6c.

Whereas the AUCs measured for the training sets remain constant at around 0.92, the AUCs obtained for the test sets confirm that we are able to generate successively harder splits, as by design. The easiest tree-based split is about as hard as the hardest cluster-based split. The 30% sequence-identity split, on the other hand, ranks among the easier splits, falling in between two cluster-based splits with thresholds t=1.6 and 3.0 (see our previous discussion around accidental train-test leakage, Fig. 5).

We think that this series of progressively harder train-test splits derived from an expressive similarity metric such as EPoCS is a meaningful but not yet well-established practice that can help construct more believable benchmarks. They provide an improved quantitative understanding of how robust and transferable pocket-centric ML models are, whether they are overly reliant on memorization, or are unstable with respect to training-set selection. It is this type of quantitative insight that helps build trust in the models that are prioritized for further development or deployment.

## 5 Conclusions

Expressive similarity metrics that capture the huge diversity of protein binding sites and their biochemical, functional, and evolutionary contexts have many important applications in target and therapeutic discovery. However, striking the right balance between multiple interrelated channels of information at different scales, from local atomic structure to global sequence, is challenging.

Here, we have introduced EPoCS, an alignment-free metric for binding-site similarity based on a state-of-the-art PLM. The approach shows great promise in capturing relevant, complex patterns at multiple scales while being computationally efficient. Combined with hierarchical clustering, the metric induces a feature-rich topological projection of the pocketome that we find aligns well with an intuitive understanding of binding-site context from structural, chemogenomic, and biochemical perspectives.

Besides enabling interactive queries on pockets of interest for binding-site exploration, EPoCS can serve as a foundation for automatic contextualization and transfer of ligand information or hit matter across (sub-)pockets and proteins. Our understanding of its applicability to nonliganded or orphan sites is, however, still incomplete, and requires further study using suitable curation and validation sets. Future research may also address to what degree EPoCS-style embeddings can augment dynamical or structural models for druggability and cryptic-site detection.

As deep-learning-based models for pocket-centric tasks such as druggability prediction, co-folding and affinity modelling gain increasing relevance, deep debiasing of train-test splits—as achieved by EPoCS and illustrated for druggability prediction—is urgently needed to construct believable benchmarks that provide fair estimates of how these memorization-prone methods perform in the real world. These debiased splits not only serve as useful feedback during development of ML models, but help deploy the right model for the right reasons.

## Supplementary Material

btaf284_Supplementary_Data

## Data Availability

The PDB subsets and clusterings are available publicly at https://github.com/tugceoruc/epocs/tree/pre-release/data.

## Code availability

Code for the generation of site embeddings, site projection, and visualization is available at https://github.com/tugceoruc/epocs.
