# Supplementary material for: Mapping the space of protein binding sites with sequence-based protein language models
Source: Bioinformatics. 2025 Jun 27;41(6):btaf284. doi: 10.1093/bioinformatics/btaf284 (PMC12208174; doi:10.1093/bioinformatics/btaf284)
Supplement: btaf284_Supplementary_Data [file btaf284_supplementary_data.pdf]

SUPPLEMENTARY INFORMATION

Mapping the space of protein binding sites with sequence-based protein language models

Tuğçe Oruç,[1] Maria Kadukova,[1] Thomas G. Davies,[1] Marcel Verdonk,[1] and Carl Poelking[1,*]
[1]*Astex Pharmaceuticals, Cambridge, UK*

Dataset construction. The cluster maps in Figs. 3 and 4 of the main text were derived from a custom subset of the PDB with 96301 structures of liganded binding sites across 68629 PDB entries. The filters that gave rise to this subset are as follows: Ligands classified as additives, stabilizers, saccharides, cofactors, ions or ion clusters were discarded [1], as were PDB structures containing nucleic or non-standard amino acids, and proteins containing chains with more than 1024 residues.

Site embeddings. Given a protein and a ligand, binding-site residues were determined with Voronoi tessellation, followed by distance-based filtering. While Voronoi tessellation indicates residues with a surface cross-section with the ligand, a radial cutoff of 8 Å between the backbone atoms and any ligand atom is used to remove distant residues.

To obtain PLM residue embeddings, the chain sequences of the PDB structures were extracted and fed as input to a pretrained ESM-2 model (esm2_t36_3B_UR50D) [2]. Given the embeddings for the whole sequence of length L (i.e., the $L \times 2560$ matrix), a ligand-biased site embedding subsequently used as pocket representation was generated with mean pooling over the binding site residues.

Clustering and visualisation. The distance matrix for hierarchical clustering was generated using the pairwise Euclidean distance between the pocket representations. The choice of Euclidean distance over cosine similarity/distance was weakly motivated by the fact that EPoCS embeddings are pooled over multiple residue vectors, with amplitude thus expected to play a marginally more important role than for the unpooled vectors. The linkage policy for the clustering was based on the minimum distance of approach between any of the members of a pair of clusters. To avoid redundancy and reduce the memory footprint, pre-clustering was applied with a small distance cutoff ($t = 1.0$) and one representative was selected from each cluster as a clustering landmark.

For the pocket maps in Figs. 3 and 4, clusters were merged up to a distance threshold of $t = 1.6$. We note that the pre-clustering step can result in violations of the distance guarantee between clusters, which is why the final distance between nearby pairs of clusters was re-evaluated to construct the effective cluster-cluster distance matrix. Edges of the Pocket Atlas were calculated from the cluster-distance matrix using a minimum spanning tree algorithm as implemented in the tmap library [3]. The interactive version of the map was constructed using the Faerun package [4].

Comparison to APoc and SeqId. We evaluated the correlation of EPoC distances with APoc [5, 6] and a simple sequence distance measure over a random subset of $\sim$ 3.5k pockets. We used the same binding-site definition for APoc and EPoCS as defined above. For each pocket pair (a, b), APoc similarity scores were calculated in both directions, a vs b and b vs a, to account for differences in normalisation, with the average of the two scores used as the final similarity s. We used $d = 1 - s$ as the associated distance value for both APoc and sequence similarity.

Clustering distance. We used optimal transport over the cluster-cluster Jaccard index to measure the similarity between the clusterings induced by EPoCS vs APoc (Table S1). We used identical clustering procedures for both the EPoCS and APoc metrics (see clustering details above). The distance thresholds for both techniques were chosen so as to approximately match the resulting number of clusters (for example, we match the APoc threshold $u = 0.2$ to an EPoCS threshold of $t = 1.6$, resulting in, respectively, 1938 vs 1916 clusters). To additionally compare to the binding-site families defined by Binding MOAD [7], which are ultimately derived from the APoc metric, we filtered the set of PDB IDs down to those present in both sets. This latter comparison was based on PDB IDs (not pockets) in line with Binding MOADs description of site families.

We calculated the Jaccard index for all pairs of clusters induced by the different metrics (i.e., the number of pockets shared by the cluster pair over the total number of pockets associated with that pair). Optimal transport [8] was applied to the cluster-cluster similarity matrix to find the best-matching cluster assignment. We use the average of the Jaccard index for the assigned clusters as similarity for the two clusterings. As a null baseline, we also report the final similarity obtained for scrambled clusterings, where the number and size distribution of the clusters is retained, but cluster membership is shuffled.

Map annotation. We used the SIFTS database [9, 10] to annotate the PDB structures with their Enzyme Commission (EC) numbers. A cluster of binding sites was labeled as belonging to a certain EC class if: at least one member of the cluster belongs to that EC class; and there is no pocket in that cluster belonging to any other class.

Active-site information was retrieved using EBI's API. Residue mappings from UniProt to the PDB were extracted from SIFTS. For a given PDB ID and EPoCS binding site, the pocket was labeled as 'active' if the mapped residue ID was among the binding-site residues.

For the annotation of the Pocket Atlas, a cluster was labeled as 'active' if all pockets included in that cluster were 'active'. For some structures, active site information is missing or not available. Clusters of pockets that are associated with activity labels but that do not incorporate the 'active' residues them-

* carl.poelking@astx.com

selves, are labeled as 'non-active'. Other clusters are grouped under the label 'mixed'.

To identify clusters with diverse sequences, pre-generated sequence-based clusters with 30% identity cutoff based on MMseqs2 [11]) were adopted from the PDB. For EPoCS clusters split across several sequence clusters, pairwise sequence alignments were generated and their pairwise sequence identities calculated. Pocket clusters associated with at least one pair of proteins with sequence identity less than 30% were labeled as sequence 'diverse'.

QED scores were calculated with RDKit [12], with the maximum QED score used to represent the cluster.

To quantify the spatial autocorrelation of the properties shown in Fig. 4, we used a kernelized form of Moran's I:

$$I = C\frac{\sum_{i=1}^{C}\sum_{j=1}^{C} W_{ij}K_{ij}}{\sum_{i=1}^{C} K_{ii}},$$

where C is the number of clusters; W and K are the coupling weight and kernel matrix, respectively, defined via

$$\begin{aligned} W_{ij} &= z^{-1}\exp\left(-\alpha d_{ij}\right), \\ K_{ij} &= \exp[-\beta(y_i - y_j)^2], \end{aligned}$$

with d_{ij} the centroid distance between clusters, drop-off constant $\alpha = 2t_2^{-1}$, normalization factor $z = \sum_{i,j} W_{ij}$, property labels y_i, y_j associated with clusters i and j, length scale $\beta = \text{Var}(y)^{-1}$. For categorical labels, we replace K_{ij} with a delta kernel, $K_{ij} = \delta(y_i - y_j)$.

Druggability modelling. The ESM-based druggability model MLP, consists of a multilayer perceptron (MLP) with residue ESM-embeddings as inputs and a single log-probability channel as output. The MLP includes a single hidden layer with 12 nodes. Binary training labels for residues were assigned based on the same Voronoi tessellation procedure as used by EPoCS. We used an Adam optimizer with a constant learning rate of 0.001 and weight decay of 0.001. The MLP's dropout rate was set to 0.3. The models were trained with a log-likelihood loss function on the 'general' set of PDBbind 2020.

Comparison to fpocket and PointSite. Despite its simplicity, ESM-MLP is found to be performant – see Figs. S2 and S3 for a comparison to fpocket and PointSite. We note, however, that training set consistency is not maintained across the three models. Hence, the structures which are present in both HOLO4K (one of the test sets of PointSite) and the 'general' PDBBind set were selected as an additional test set that allows for a direct comparison between PointSite and ESM-MLP.

Unlike the ESM-based model, fpocket does not assign continuous druggability scores to individual residues. We therefore used membership labels of the residues belonging to a predicted binding site as a binary proxy for an fpocket druggability score. PointSite, on the other hand, produces atomic druggability scores, which we translated into residue-level scores using max pooling. For PointSite, fpocket, and ESM-MLP, cluster AUCs were evaluated by concatenating the modelled residue scores and calculating the true-positive and false-positive rates over the ligand-based ground-truth contact labels as a function of score threshold.

Software. We used pdb-tools [13], pymol [14] and biopandas [15] to process PDB structures, and RDKit for molecular processing. Hierarchical clustering was performed using SciPy [16]. Optimal transport metrics were evaluated using the POT package [8].

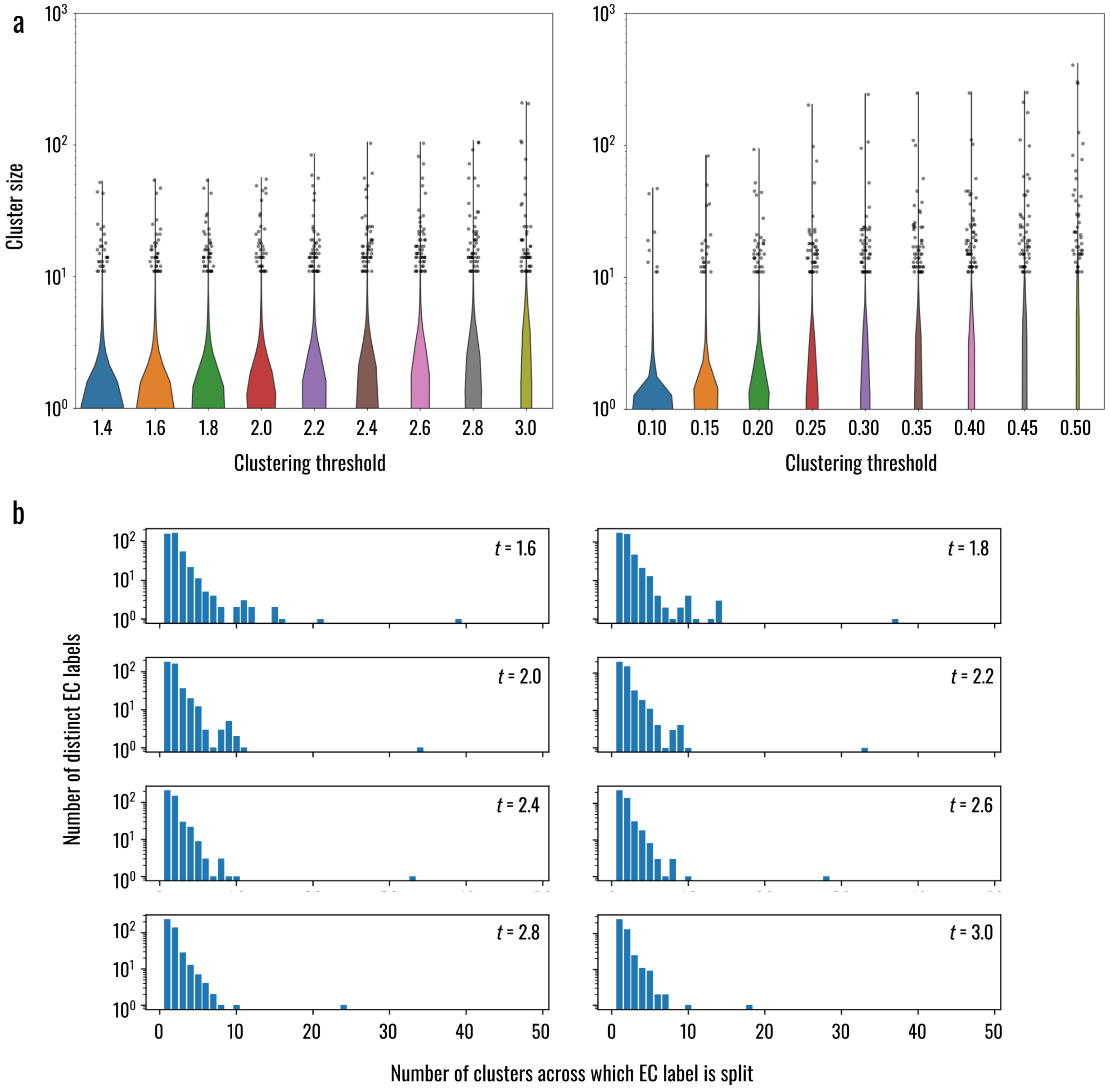

Figure S1. **Clustering statistics at different separation thresholds.** (a) Violin plots of the cluster size distribution induced by (left) EPoCS and (right) APoc for several clustering thresholds. (b) Degree of fragmentation of EC groups across EPoCS clusters at different clustering thresholds. The vast majority of EC groups are contained within fewer than 10 clusters, indicating that the clustering outcome is well aligned with functional annotations over the range of thresholds explored here.

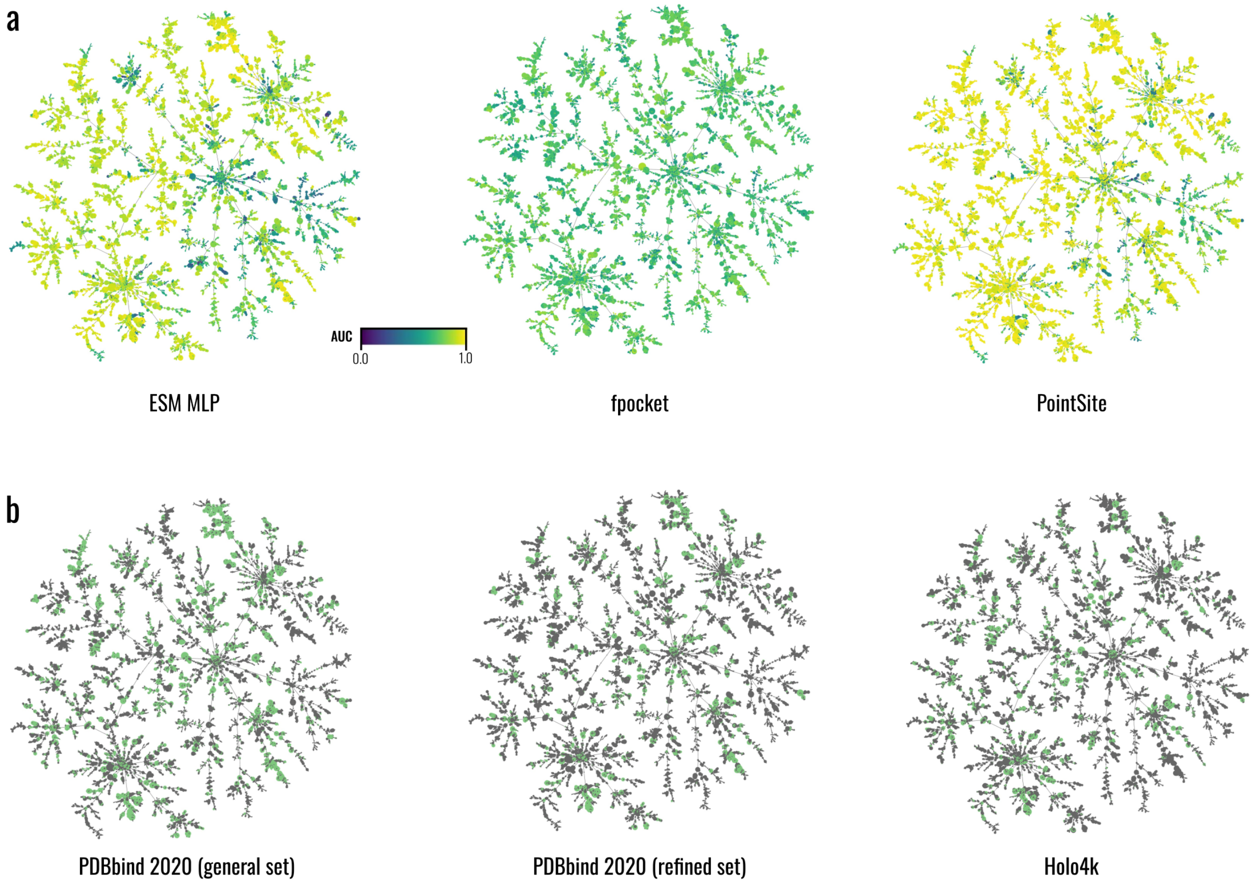

Figure S2. **Comparison of druggability prediction performance across the EPoCS atlas.** (a) Per-cluster AUCs assessing the predicted per-residue druggability scores generated by the ESM MLP, fpocket and PointSite vs the ground-truth contact labels. Despite its simplicity, the ESM MLP rivals PointSite in the quality of its predictions, with fpocket-based proxy scores falling behind the other two methods. Interestingly, the ESM MLP and PointSite are broadly aligned in how their AUCs vary across the pocket atlas, indicating a similar concept of druggability learnt by these two models. These global trends are less perceptible in the case of fpocket, which is a volume-based rather than contact-based approach. (b) Distribution of PDBbind 2020 subsets used in the training and evaluation of the ESM MLP across the pocket atlas.

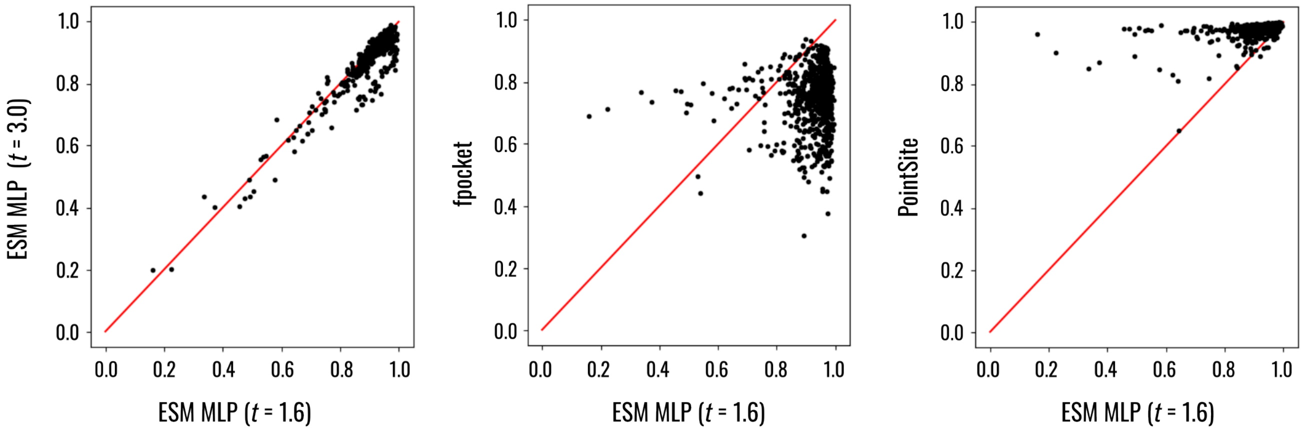

Figure S3. **Score correlations on the intersection of HOLO4K and PDBbind.** Score correlations among four druggability models (ESM MLP trained on sets derived from two different EPoCS thresholds $t = 1.6, 3.0$, fpocket and PointSite. HOLO4K is part of PointSite's test set, making this a fair comparison between the ESM MLP models and PointSite.

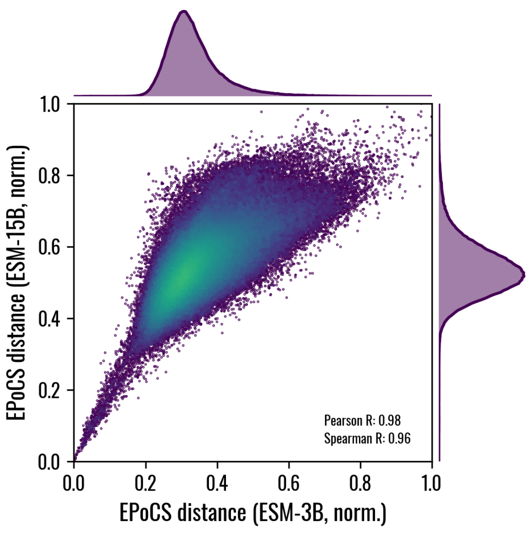

Figure S4. **Correlation between metric spaces induced by different protein language models.** Site-site distance correlation between EPoCS with ESM2-3B vs EPoCS with ESM2-15B. The comparison set is PDB-3.5k (see main text).

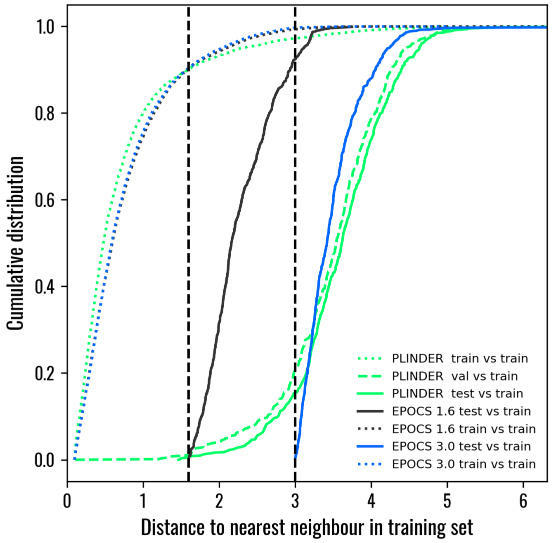

Figure S5. **Comparison between PLINDER and EPoCS splits.** Distributions of the EPoCS distance of test set samples to their nearest training sample for EPoCS and PLINDER splits [17]. The splits are defined relative to the PLINDER set of protein-ligand structures, where the PLINDER split analysed here corresponds to version 2024-06/v2. We note that the distance distribution of the EPoCS split with threshold $t_2 = 3.0$, despite being based on an alignment-free approach, is qualitatively and quantitatively similar to the distance distribution measured for the PLINDER split. The onset of the PLINDER distribution begins at an EPoCS distance of 1.6, which happens to coincide with the lower EPoCS clustering threshold and might indicate minor train-test leakage.

Figure 3

PDB code	EC label	System description
4dcn	-.-.-.-	ADP-ribosylation factor-like protein 1
6bbq	3.6.5.2	Cytohesin-3, ADP-ribosylation factor 6
1aha	3.2.2.22	Cytohesin-3, ADP-ribosylation factor 6
2pqj	3.2.2.22	Ribosome-inactivating protein 3
4dki	3.4.16.4	Penicillin-binding protein 2’
1mwu	2.4.1.129	Penicillin-binding protein 2a
3d4a	3.1.4.8	Ribonuclease
2ya7	3.2.1.18	Sialidase A, Neuraminidase A
3h73	3.2.1.18	Sialidase A, Neuraminidase A
2c6z	3.5.3.18	N(G)-dimethylarginine dimethylaminohydrolase 1
2jaj	3.5.3.18	N(G)-dimethylarginine dimethylaminohydrolase 1
2yis	2.7.11.24	Mitogen-activated protein kinase 14
2yiw	2.7.11.24	Mitogen-activated protein kinase 14
3c5u	2.7.11.24	Mitogen-activated protein kinase 14
4eh7	2.7.11.24	Mitogen-activated protein kinase 14
5n65	2.7.11.24	Mitogen-activated protein kinase 14
2xcw	3.1.3.5	Cytosolic purine 5’-nucleotidase
2xje	3.1.3.5	Cytosolic purine 5’-nucleotidase
2xjc	3.1.3.5	Cytosolic purine 5’-nucleotidase

Figure 5

PDB code	EC label	System description
8slg	6.1.1.14	Glycine–tRNA ligase
4hwo	6.1.1.3	Threonine–tRNA ligase
7cij	4.1.1.57	L-methionine decarboxylase
5w19	4.1.99.1	Tryptophanase, L-tryptophan indole-lyase, TNase
7sza	3.6.-.-	EKC/KEOPS complex subunit TP53RK
6hk6	2.7.11.1	Serine/threonine-protein kinase RIO2
7ljn	2.7.7.85	Cyclic dinucleotide synthase CdnG
4u03	2.7.7.86	Cyclic GMP-AMP synthase

Table S1. PDB codes referenced in Figs. 3 and 5 of the main text, with EC label and system description.

[1] Feng, Z. *et al.* Ligand Depot: a data warehouse for ligands bound to macromolecules. *Bioinformatics* **20**, 2153–2155 (2004). URL https://doi.org/10.1093/bioinformatics/bth214. https://academic.oup.com/bioinformatics/article-pdf/20/13/2153/48905838/bioinformatics_20_13_2153.pdf.

[2] Lin, Z. *et al.* Evolutionary-scale prediction of atomic-level protein structure with a language model. *Science* **379**, 1123–1130 (2023). URL https://www.science.org/doi/abs/10.1126/science.ade2574. https://www.science.org/doi/pdf/10.1126/science.ade2574.

[3] tmap. URL https://github.com/reymond-group/tmap.

[4] faerun. URL https://github.com/reymond-group/faerun.

[5] Gao, M. & Skolnick, J. APoc: large-scale identification of similar protein pockets. *Bioinformatics* **29**, 597–604 (2013). URL https://doi.org/10.1093/bioinformatics/btt024. https://academic.oup.com/bioinformatics/article-pdf/29/5/597/50335709/bioinformatics_29_5_597.pdf.

[6] apoc. URL https://sites.gatech.edu/cssb/apoc/.

[7] Smith, R. D. *et al.* Updates to binding moad (mother of all databases): Polypharmacology tools and their utility in drug repurposing. *Journal of Molecular Biology* **431**, 2423–2433 (2019). URL https://www.sciencedirect.com/science/article/pii/S0022283619302967. Computation Resources for Molecular Biology.

[8] Flamary, R. *et al.* Pot: Python optimal transport. *Journal of Machine Learning Research* **22**, 1–8 (2021). URL http://jmlr.org/papers/v22/20-451.html.

[9] Velankar, S. *et al.* SIFTS: Structure Integration with Function, Taxonomy and Sequences resource. *Nucleic Acids Research* **41**, D483–D489 (2012). URL https://doi.org/10.1093/nar/gks1258. https://academic.oup.com/nar/article-pdf/41/D1/D483/3710597/gks1258.pdf.

[10] Dana, J. M. *et al.* SIFTS: updated Structure Integration with Function, Taxonomy and Sequences resource allows 40-fold increase in coverage of structure-based annotations for proteins. *Nucleic Acids Research* **47**, D482–D489 (2018). URL https://doi.org/10.1093/nar/gky1114. https://academic.oup.com/nar/article-pdf/47/D1/D482/27437256/gky1114.pdf.

[11] Steinegger, M. & Söding, J. Mmseqs2 enables sensitive protein sequence searching for the analysis of massive data sets. *Nature Biotechnology* **35**, 1026–1028 (2017). URL https://doi.org/10.1038/nbt.3988.

[12] Rdkit: Open-source cheminformatics. URL https://www.rdkit.org.

[13] Rodrigues, J., Teixeira, J., Trellet, M. & Bonvin, A. pdb-tools: a swiss army knife for molecular structures [version 1; peer review: 2 approved]. *F1000Research* **7** (2018).

[14] Schrödinger, LLC. The PyMOL molecular graphics system, version 1.8 (2015).

[15] Raschka, S. Biopandas: Working with molecular structures in pandas dataframes. *Journal of Open Source Software* **2**, 279 (2017). URL https://doi.org/10.21105/joss.00279.

[16] Virtanen, P. *et al.* SciPy 1.0: Fundamental Algorithms for Scientific Computing in Python. *Nature Methods* **17**, 261–272 (2020).

[17] Durairaj, J. *et al.* Plinder: The protein-ligand interactions dataset and evaluation resource (2024). URL https://www.biorxiv.org/content/10.1101/2024.07.17.603955v1.
